# The Intention to Conceal Does Not Always Affect Time Perception

**DOI:** 10.3389/fpsyg.2021.781685

**Published:** 2021-12-10

**Authors:** Izumi Matsuda, Hiroshi Nittono

**Affiliations:** ^1^Department of Psychology, Aoyama Gakuin University, Shibuya, Japan; ^2^Graduate School of Human Sciences, Osaka University, Suita, Japan

**Keywords:** time perception, concealed information test, arousal, online experiment, physiological index

## Abstract

The display duration of stimuli is overestimated due to the increase in phasic arousal induced by the stimuli or high levels of background arousal. A previous study demonstrated that display duration of items (2 s) was overestimated when a participant attempted to conceal one of the items so as not to be detected in the concealed information test (CIT). As the time perception remained the same between the item to be concealed and the other items, the overestimation was thought to be due to the high level of background arousal under the conceal condition. Duration of 2 s may be too long to examine the phasic arousal effect induced by the concealed item. The present study conducted three online experiments with shorter durations, that is, each of three items was presented with duration of 1, 0.5, and 2 s in Experiments 1, 2, and 3, respectively. The participants were instructed to conceal one of the three items under the conceal condition and did not conceal any item in the innocent condition. The difference in time perception between the conceal and innocent conditions or between items under the conceal condition was observed in none of the three experiments. The result indicates that temporal overestimation does not occur when a participant is only concealing an object. Rather, temporal overestimation would occur only when the level of background arousal is amplified by the concealment.

## Introduction

The fact that arousal speeds up the pacemaker of an internal clock is well known (Treisman, 1963; Gibbon et al., 1984; Zakay and Block, 1997; Droit-Volet and Meck, 2007). The display durations of stimuli are generally overestimated when the level of background arousal is high under certain conditions (Wittmann and Paulus, 2008; Droit-Volet et al., 2011; Piovesan et al., 2019). The duration of a stimulus is also overestimated when it induces the increase of phasic arousal compared with non-arousing stimuli (Gil and Droit-Volet, 2012).

Arousal change occurs under various cognitive processes, one of which is concealment. Previous scholars have examined the psychological processes related to concealment using the paradigm of the concealed information test (CIT), which is a psychological tool for criminal investigations. The CIT presents one relevant item that the guilty person should be aware of and intend to conceal embedded among other irrelevant items. Previous studies on the CIT indicate that the relevant item elicits orienting responses that induce phasic arousal (e.g., increased skin conductance response) and responses related to cognitive control to inhibit the orienting responses (e.g., respiration suppression; Matsuda et al., 2013; klein Selle et al., 2016, 2018; Matsuda and Nittono, 2018). Due to the phasic arousal increase, the duration of the relevant item in the CIT is expected to be perceived as longer than those of irrelevant items.

Matsuda et al. (2020) investigated time perception during the CIT. In their experiment, the participants were instructed to steal an item and conceal it during the experiment. Each participant was presented with two conditions. The first includes pictures of three items, including the stolen one (i.e., conceal condition), whereas the second uses pictures of three items that were not stolen (i.e., innocent condition). The participants were instructed to determine the display duration of each picture as shorter than, equal to, or longer than a set duration of 2 s. The items were consistently presented for 2 s. However, the display duration of the items was perceived as longer under the conceal condition than that under the innocent condition. Matsuda et al. explained that this disposition was caused by an increased level of arousal when the participants were concealing an item, as evidenced by higher levels of skin conductance under the conceal condition than that under the innocent condition.

Contrary to the expectation, Matsuda et al. (2020) did not determine the difference in time perception between the relevant item to be concealed and the other irrelevant items under the conceal condition, despite the increased skin conductance response for the relevant item than the irrelevant items. Gil and Droit-Volet (2012) suggested that the effect of phasic arousal induced by a stimulus on time perception decreased when the stimulus duration exceeded 1 s. The authors argued that subjective time distortions with brief durations (i.e., less than 1 s) are a result of the action of a pure arousal mechanism. However, the interference between arousal and attention occurs with longer durations, which may reduce the time distortion phenomenon (Coull et al., 2004). The null finding of Matsuda et al. (2020) between items may be due to the longer stimulus duration of 2 s.

The present study aims to confirm whether the item to be concealed would be perceived as longer than the other items using shorter display durations than those used in Matsuda et al. (2020). Experiment 1 used a display duration of 1 s instead of 2 s to observe the stimulus-induced arousal effect (Gil and Droit-Volet, 2009). We then conducted Experiments 2 and 3, whose protocols were the same as those of Experiment 1 but with different durations. In Experiment 2, we used a shorter duration of 0.5 s given ERP studies that stated that cognitive control-related processes to inhibit arousal would occur at approximately 0.5 s after the onset of the item (Matsuda et al., 2013; Matsuda and Nittono, 2018). In Experiment 3, we used duration of 2 s to replicate Matsuda et al. (2020). The study presents the following hypotheses.

H1: The display duration of the items is perceived as longer in the conceal condition than in the innocent condition in all experiments.

H2: The display duration of the relevant item to be concealed is perceived as longer than that of the other irrelevant items in the conceal condition in Experiment 1 (item duration = 1 s) and 2 (item duration = 0.5 s) but not in Experiment 3 (item duration = 2 s).

The main experimental protocol of the present study was the same as that of Matsuda et al. (2020) except for the following points. First, although the previous study conducted experiment face-to-face, the present study conducted all experiments online. Thus, the participants did not meet the experimenter and undertook the CIT alone without the measurement of physiological indices. Several studies have shown that online CITs are feasible. They produced similar results to those obtained in traditional face-to-face CITs based on reaction times (Verschuere and Kleinberg, 2016; Lukács et al., 2017; Lukács and Ansorge, 2019). Second, although the participants memorized the object through a mock theft in the previous study, they memorized the object on the card they selected in the present study. A meta-analysis of the CIT (Ben-Shakhar and Elaad, 2003) showed that even when the participants memorized the relevant item without performing a mock crime, the relevant item elicited greater arousal responses than the irrelevant items. Therefore, the attempt to conduct the current experiments online with a method other than a mock crime is acceptable.

## Materials and Methods

### Participants

The effect size of H1 in Matsuda et al. (2020) was *d*z = 0.436. To detect this effect with a power of 0.95 by a two-tailed *t*-test (*p* < 0.05), a sample size of 71 estimated by G*Power (ver. 3.1.9.2; Faul et al., 2007) was considered adequate. The study recruited participants through a crowdsourcing company (CrowdWorks, Japan) for each experiment. Those met the exclusion criteria (section “Exclusion Criteria”) were excluded. The remaining participants were 72 [30 men and 42 women; *M* = 38.85, standard deviation (*SD*) = 7.85] for Experiment 1; 73 (39 men and 34 women; *M* = 39.05, *SD* = 8.60) for Experiment 2; and 71 (31 men and 40 women; *M* = 41.66, *SD* = 9.14) for Experiment 3. The Ethics Committee of Aoyama Gakuin University approved the study (approval number: AO20-16).

### Stimuli

The stimuli were the same as those used in Matsuda et al. (2020). Two stimulus sets of real objects were prepared. The first consisted of three accessories (a ring, a necklace, and earrings), whereas the second consisted of three electronic products (a mobile phone, a digital camera, and a voice recorder). A photograph of each object was taken and presented in three angles (i.e., upright, left-rotated, and right-rotated) for a total of nine pictures per set (3 objects × 3 angles).

### Procedure

The experiment was created using Inquisit 6 and the participants conducted it online via Inquisit Web Player 6.3.5 or 6.3.4.^1^ The participants conducted the experiment using their PC and received 440 JPY as compensation (equivalent to approximately 4 USD) if they successfully reached the end of the experiment.

**FIGURE 1:**
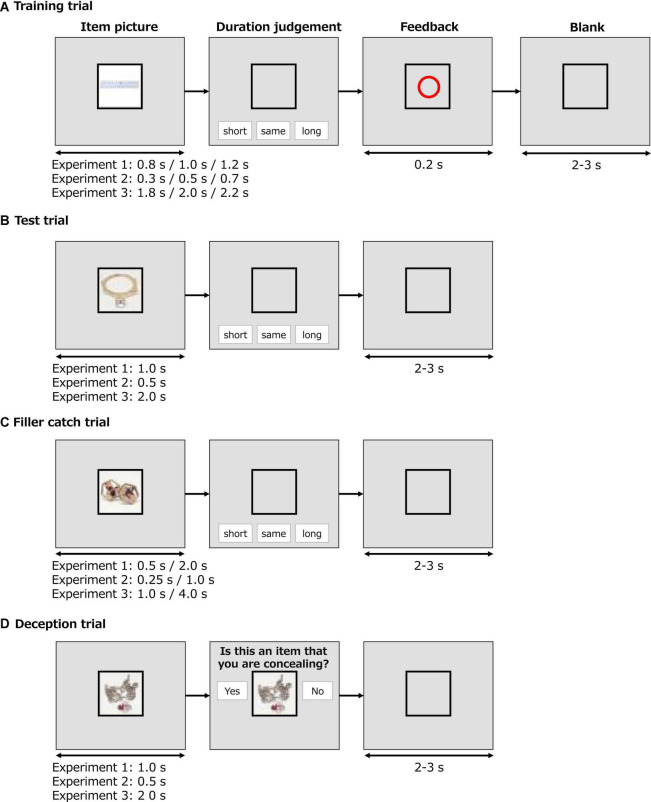
Examples of a trial in the training session and a test trial, a filler catch trial, and a deception trial in the test session. **(A)** Training trial. **(B)** Test trial. **(C)** Filler catch trial. **(D)** Deception trial.

First, the participants memorized standard durations of 1, 0.5, and 2 s in Experiments 1, 2, and 3, respectively, by looking at pictures for nine times. These pictures were three stationaries and would not be used in the test session. The standard duration was not explicitly stated. Then, the training session began (see Figure 1A). In training trials, the same pictures were randomly presented for the standard duration and standard duration ± 0.2 s (i.e., 0.8, 1.0, or 1.2 s in Experiment 1; 0.3, 0.5, or 0.7 s in Experiment 2; and 1.8, 2.0, or 2.2 s in Experiment 3). After displaying the picture, buttons labeled as “short,” “equal,” or “long” appeared on the screen. The participants decided whether the duration was “short,” “equal,” or “long” compared with the memorized standard duration. To avoid confusion, the training session started with three trials by presenting a picture with a standard duration. If they responded correctly in two out of three trials, then the randomized training trials started. If not, then three more trials with the standard duration were repeated. The intertrial interval was 2–3 s, and the training session was completed when the correct response rate exceeded 80% in the last five randomized training trials.

The participants were then asked to memorize an object. They were instructed to select one of six cards on the screen. The selected card was turned over and a picture of an object (i.e., ring, necklace, earrings, mobile phone, digital camera, or voice recorder) was presented. The participants should remember and conceal the object until the end of the experiment. Unbeknownst to the participants, the object to be memorized was counterbalanced across participants.

In the test session, shown as a test trial in Figure 1B, each picture of a stimulus set was presented three times on the screen with a standard display duration (3 objects × 3 angles × 3 times = 27 trials in random order). Figure 1C presents a filler catch trial in which each of the three upright pictures was presented for half (0.5 s in Experiment 1; 0.25 s in Experiment 2; 1 s in Experiment 3) or twice (2 s in Experiment 1; 1 s in Experiment 2; 4 s in Experiment 3) of the standard duration (3 objects × 1 angle × 2 durations = 6 trials). Once a picture disappeared from the screen, “short,” “equal,” and “long” buttons appeared. The participants decided whether the display duration was “short,” “equal,” or “long” compared to the memorized standard duration. In addition to these trials, a deception trial (Figure 1D) was inserted infrequently in which the question “Is this an item that you are concealing?” was presented with “yes” and “no” buttons after the display of each of the three upright pictures (3 objects × 1 angles × 2 times = 6 trials) to remind the participants that they had to conceal the chosen object. The participants were expected to press the “no” button to all items. In total, 39 trials were conducted in random order with an intertrial interval of 2–3 s. No speeded response was required.

The abovementioned test session was repeated using the other stimulus set. The test session using a stimulus set that included an item to be concealed was defined as the conceal condition, whereas the other test session was defined as the innocent condition. The order of these conditions was counterbalanced across participants. The item to be concealed was defined as the relevant item in the conceal condition, whereas the sham “relevant” item in the innocent condition was counterbalanced across participants.

Lastly, the participants rated each of the six objects on two scales: stimulus valence (from 1 = *extremely unpleasant* to 7 = *extremely pleasant*) and motivational direction (from 1 = *extremely want to avoid* to 7 = *extremely want to approach*). They were then instructed to select the object they memorized at the beginning of the experiment from the pictures of the six objects. In addition, they rated how well they concealed the object during the experiment using a seven-point scale (from 1 = *not at all successful* to 7 = *extremely successful*).

### Exclusion Criteria

The participants who met either of the following conditions were excluded from each of the three experiments.

1. Those who failed to recall the object they preselected at the end of the experiment.
2. Those who failed in more than two out of the six filler catch trials in either of the two conditions.
3. Those who failed in more than one out of the six deception trials in either of the two conditions.

### Analysis

The frequencies of the “short,” “equal,” and “long” responses were counted separately for each item in each condition, and the mean index of time judgment was calculated as follows: (number of “long” responses − number of “short” responses)/total number of responses (Mella et al., 2011). The index ranges from −1 to + 1. A positive value indicates overestimation, whereas a negative value indicates underestimation of temporal duration. For the irrelevant items, the values for the two items were averaged.

The time judgment index and subjective ratings (stimulus valence and motivational direction) were subjected to a two-way analysis of variance (ANOVA) (Condition [conceal or innocent] × Item [relevant or irrelevant]) with repeated measures for each of the three experiments. The effect sizes were described as partial η^2^ (η*_*p*_*^2^) for ANOVA and Cohen’s *d* for *t*-tests. We focused on the main effect of condition and the interaction effect to verify H1 and H2, respectively. When testing for the difference between two means, Bayes factor is computed using JASP 0.14.1.^2^ Lastly, a three-way ANOVA (Duration [1 s, 0.5 s, or 2 s] × Condition × Item) was conducted for confirmation, because the three experiments were very similar except for stimulus duration.

The abovementioned protocols were registered using duration of 1 s (i.e., Experiment 1). The preregistered protocol, stimulus materials, and obtained data are available at https://osf.io/m2zeg/.

## Results

To confirm whether the participants tried to conceal their knowledge, we first checked the subjective rating of how well they concealed the memorized object. The average ratings were 5.99 (*SD* = 1.26, range = 2–7), 6.06 (*SD* = 1.24, range = 3–7), and 5.94 (*SD* = 1.26, range = 2–7) for Experiments 1, 2, and 3, respectively. In other words, the majority of the participants felt that they could conceal the memorized object.

### Time Judgment Index

**FIGURE 2:**
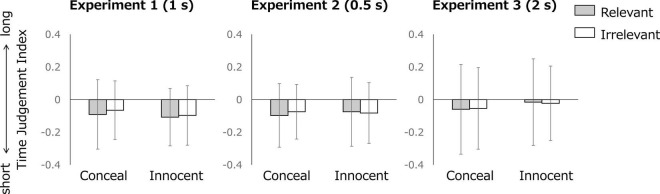
The mean time judgment index for each condition and each item. Error bars denote standard deviations.

Figure 2 presents the mean time judgment index for each condition and each item in Experiments 1, 2, and 3. Table 1 shows the results of Condition × Item ANOVA related to H1 (main effect of condition) and H2 (interaction). In all experiments, neither of the main effects of condition or interactions was significant. Table 1 also depicts the results of Duration × Condition × Item ANOVA, which indicated that neither of the main effect of condition or the Condition × Item interaction was significant. Other results for Duration × Condition × Item ANOVA indicate no significant effects of duration [main effect of duration: *F*(2, 213) = 1.929, *p* = 0.148, η*_*p*_*^2^ = 0.018; Duration × Condition interaction: *F*(2, 213) = 1.942, *p* = 0.146, η*_*p*_*^2^ = 0.018; Duration × Item interaction: *F*(2, 213) = 0.521, *p* = 0.275, η*_*p*_*^2^ = 0.005; Duration × Condition × Item interaction: *F*(2, 213) = 0.103, *p* = 0.902, η*_*p*_*^2^ < 0.001]. As an exploratory analysis, the same ANOVAs were conducted separately for men and women. However, the results did not change and no significant differences in the time judgment index were found (see Supplementary Material).

**TABLE 1:** Results of the Condition × Item ANOVA in each experiment and Duration × Condition × Item ANOVA across all experiments.

	Main effect of condition (H1)				Condition × Item (H2)			
	*F*	*p*	η_p_^2^	BF_10_	*F*	*p*	η_p_^2^	BF_10_
**Time judgment index**								
Experiment 1 [1 s, *df* = (1, 71)]	1.728	0.193	0.024	0.295	0.294	0.589	0.004	0.149
Experiment 2 [0.5 s, *df* = (1, 72)]	0.195	0.660	0.003	0.141	0.956	0.331	0.013	0.204
Experiment 3 [2 s, *df* = (1, 70)]	1.654	0.203	0.023	0.287	0.168	0.683	0.002	0.141
All experiments [*df* = (1, 213)]	0.282	0.596	0.001	0.087	1.257	0.263	0.006	0.143
**Subjective stimulus valence**								
Experiment 1 [1 s, *df* = (1, 71)]	3.323	0.073	0.045	0.621	1.117	0.294	0.015	0.221
Experiment 2 [0.5 s, *df* = (1, 72)]	5.506	0.022	0.071	1.668	11.232	0.001	0.135	19.977
Experiment 3 [2 s, *df* = (1, 70)]	3.644	0.060	0.049	0.724	2.526	0.116	0.035	0.432
All experiments [*df* = (1, 213)]	11.689	0.001	0.052	21.864	10.358	0.001	0.046	11.662
**Subjective motivational direction**								
Experiment 1 [1 s, *df* = (1, 71)]	0.831	0.365	0.012	0.193	0.038	0.846	0.001	0.132
Experiment 2 [0.5 s, *df* = (1, 72)]	1.841	0.179	0.025	0.309	2.929	0.091	0.039	0.515
Experiment 3 [2 s, *df* = (1, 70)]	3.834	0.054	0.052	0.789	2.130	0.149	0.030	0.359
All experiments [*df* = (1, 213)]	5.872	0.016	0.027	1.330	3.667	0.057	0.017	0.462

*Results related to H1 and H2 are extracted.*

### Subjective Stimulus Valence

**FIGURE 3:**
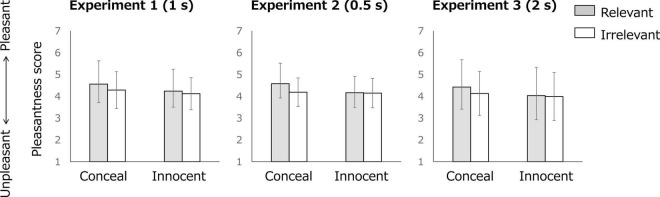
Subjective stimulus valence for each condition and each item. Error bars denote standard deviations (*SD*s).

Figure 3 depicts the mean subjective rating of stimulus valence for each condition and each item in Experiments 1, 2, and 3. Table 1 presents the results of Condition × Item ANOVA related to H1 (main effect of condition) and H2 (interaction). The main effect of condition was significant for Experiment 2, whereas it is marginally significant for Experiments 1 and 3. The Duration × Condition × Item ANOVA demonstrated that both of the main effect of condition and the Condition × Item interaction were significant. The items were evaluated as more pleasant in the conceal condition (*M* = 4.31) than in the innocent condition (*M* = 4.10). Moreover, the relevant item was evaluated as more pleasant than the irrelevant items in the conceal condition [*t*(215) = 4.832, *p* < 0.001, *d* = 0.327] but not in the innocent condition [*t*(215) = 1.143, *p* = 0.254, *d* = 0.068]. No significant effects of duration were observed [main effect of duration: *F*(2, 213) = 1.264, *p* = 0.285, η*_*p*_*^2^ = 0.012; Duration × Condition interaction: *F*(2, 213) = 0.028, *p* = 0.972, η*_*p*_*^2^ < 0.001; Duration × Item interaction: *F*_(2, 213)_ = 0.059, *p* = 0.943, η*_*p*_*^2^ < 0.001; Duration × Condition × Item interaction: *F*_(2, 213)_ = 0.613, *p* = 0.543, η*_*p*_*^2^ = 0.006].

### Subjective Motivational Direction

**FIGURE 4:**
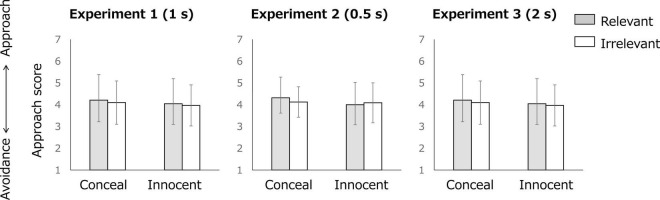
Subjective motivation direction for each condition and each item. Error bars denote standard deviations.

Figure 4 shows the mean subjective rating of motivation direction for each condition and each item in Experiments 1, 2, and 3. Table 1 shows the results of Condition × Item ANOVA related to H1 (main effect of condition) and H2 (interaction). In all experiments, neither of the main effects of condition or interactions was significant. The Duration × Condition × Item ANOVA indicated that the main effect of condition was significant. No significant effects of duration were observed [main effect of duration: *F*(2, 213) = 0.223, *p* = 0.801, η*_*p*_*^2^ = 0.002; Duration × Condition interaction: *F*(2, 213) = 0.313, *p* = 0.732, η*_*p*_*^2^ = 0.003; Duration × Item interaction: *F*(2, 213) = 1.162, *p* = 0.315, η*_*p*_*^2^ = 0.011; Duration × Condition × Item:[*F*(2, 213) = 0.619, *p* = 0.539, η*_*p*_*^2^ = 0.006].

### Individual Data of Time Judgment Index

**FIGURE 5:**
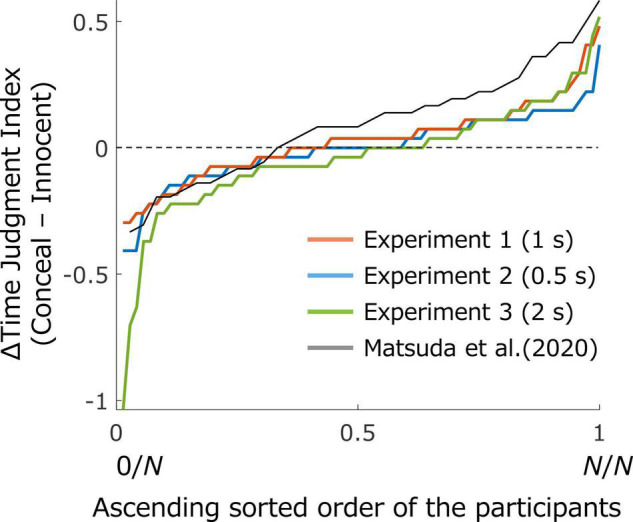
Individual data of the difference in the time judgment index between conditions. The difference was computed between the mean of the Conceal condition and that of the Innocent condition for each participant, which was organized in ascending order. The horizontal axis indicates (ascending order of each participant)/(total number of the participants), whereas the vertical axis indicates the participant’s difference in the mean between conditions. Data from Experiments 1 (blue), 2 (red), and 3 (green), and the previous study (gray) were plotted.

We computed differences in the mean of the time judgment index between the conceal and innocent conditions for each participant and categorized these differences in ascending order. Figure 5 indicates the individual data of the differences in Experiments 1, 2, and 3 and Matsuda et al. (2020), respectively. The range and the variation of the differences between the conditions were similar for the previous and present studies except for Experiment 3. However, a larger number of participants showed a positive value in the previous study than in the present study: 66.67% of the participants exceeded zero in the previous study, whereas 56.94, 41.10, and 36.62% of the participants exceeded zero in Experiments 1, 2, and 3, respectively.

## Discussion

The present study examined the effect of concealment on time perception by revising a previous study (Matsuda et al., 2020). The previous study illustrated that the overall temporal overestimation would occur under the condition in which the participants were instructed to conceal an item. However, it did not determine the temporal overestimation for the item to be concealed compared with other items. The present study used shorter item durations of 1 and 0.5 s in addition to the 2 s used in the previous study with the expectation that the effect of the increase in phasic arousal elicited by the item to be concealed would be observed with shorter durations. Contrary to the expectation, the present study did not find any difference in time perception between conditions or between items. The variations of the differences in time perception between conditions were similar between the previous and present studies. However, the overestimation effect of concealment was observed in a larger number of participants in the previous study compared to the present study. The present study indicates that time perception may not always be distorted when participants are concealing an item.

There are several potential reasons that produced these differences. Although the main experiment protocol related to concealment was the same between the previous and present studies, the present study was conducted online, whereas the previous study was conducted face-to-face. In the previous study, the experimenter was observing the participant’s responses throughout the test, of which the participant was aware. In contrast, in the present study, the participant conducted the test alone and did not meet with the experimenter. They were never aware of the existence of the experimenter or the observer during the test. Ogawa et al. (2007) proposed that the existence of the observer amplifies the skin conductance level, which reflects arousal level, during the CIT. Matsuda et al. (2020) also stated that the skin conductance level was higher in the conceal condition than in the innocent condition. In the present study, arousal level during the conceal condition may not be amplified due to the absence of the experimenter. This situation may negate temporal overestimation under the concealment. Although previous studies have shown that online CITs are feasible in that significant reaction-time differences between relevant and irrelevant items are observed (Verschuere and Kleinberg, 2016; Lukács et al., 2017; Lukács and Ansorge, 2019), online experiments may not be adequate to elevate arousal level as much as laboratory experiments.

Furthermore, in the previous study, the participant memorized the relevant item by stealing it as a mock crime, whereas the participant in the present study was only instructed to memorize the item displayed on the screen. The fact that the relevant item elicited greater physiological responses than the irrelevant items even when the participants were only asked to memorize the relevant item (i.e., without performing a mock crime) is well known (as a meta-analysis, see Ben-Shakhar and Elaad, 2003). In contrast, Elaad (2014) mentioned that the skin conductance level during the test was greater when the participants encountered the relevant item through a mock crime than when they only memorized the relevant item without performing a mock crime. In the present study, arousal level during the conceal condition would not increase due to the lack of a mock crime, which would decrease the difference in time perception between the conceal and innocent conditions.

Although we expected that the difference in time perception would be observed between the relevant and irrelevant items in the conceal condition using short display durations (i.e., 1 s and 0.5 s), no difference was found for any durations. Many previous studies have shown that the relevant item typically elicits greater skin conductance response than irrelevant items when the participants were asked to only memorize the relevant item (Ben-Shakhar and Elaad, 2003; Matsuda et al., 2006). Thus, the relevant item in the present study would also elicit increased phasic arousal than irrelevant items. The lack of difference in time perception between items may be explained by an unexpected result of subjective evaluation that the relevant item was evaluated as pleasant compared with the other items in the present study, contrary to the finding of Matsuda et al. (2020) where the relevant item was evaluated as unpleasant compared with the other items. In general, arousing negative stimuli can induce temporal overestimation, whereas arousing positive stimuli induce temporal underestimation (Gable and Poole, 2012) or no temporal distortion (Ogden et al., 2019). In the present study, the positive subjective evaluation of the relevant item may cancel the temporal overestimation elicited by the increase in item-induced arousal.

Why was the item to be concealed evaluated as pleasant in the present study? Generally, an owned object is valued higher than the same object that lacks an assigned ownership (Thaler, 1980; Kahneman et al., 1990; Morewedge and Giblin, 2015). This endowment effect is not confined to private goods, such that people value information they own more than information they do not own (Rafaeli and Raban, 2003). Thus, in the CIT, relevant information would be originally evaluated as valuable and pleasant compared with other information. In the face-to-face CIT, however, the participant is conscious of the experimenter or observer from whom the participant has to conceal knowledge. Only when the participant is aware of the other person that is observing his/her responses can the participant be motivated to avoid detection of the information owned. In this situation, information would become evaluated as unpleasant and should be avoided.

The limitation of the present study is that we did not measure physiological indices reflecting arousal, such as skin conductance level, because it was conducted online. Based on previous studies, we speculate that the present results were caused by the lack of increase in arousal level, which may be due to the absence of the observer and performance of a mock crime. However, we cannot provide a direct evidence of this notion because we did not measure any arousal indices. To examine the effect of an experimental manipulation that will influence time perception through an increase in arousal (e.g., concealment), we need to check whether the arousal is in fact amplified by measuring physiological indices such as skin conductance or at least by using a questionnaire (e.g., State-Trait Anxiety Inventory: STAI). Doing so can elucidate the discussion on the effect of experimental manipulation on time perception and can prevent mismatch of findings between studies.

## Conclusion

Matsuda et al. (2020) demonstrated that a display duration of 2 s for each item is perceived as longer when the participants are concealing one of the items. In the present study, we conducted three online experiments with display durations of 1, 0.5, and 2 s for items in the conceal condition in which the item to be concealed was presented and in the innocent condition in which the item to be concealed was not presented. In all experiments, in contrast to the previous study, the duration of the items was not perceived as longer in the conceal condition than in the innocent condition. Furthermore, similar to the previous study, the display duration of the item to be concealed was not perceived as longer than that of the other items in the conceal condition. The present study indicates that temporal overestimation may not always occur when concealing objects but may occur only when the level of background arousal is amplified with the concealment. Arousal elicited by the concealment, instead of concealment itself, would influence time perception.

## Data Availability Statement

The datasets presented in this study can be found in online repositories. The names of the repository/repositories and accession number(s) can be found below: https://osf.io/m2zeg/.

## Ethics Statement

The studies involving human participants were reviewed and approved by the Ethics Committee of Aoyama Gakuin University. The patients/participants provided their written informed consent to participate in this study.

## Author Contributions

IM and HN conceived the study and conducted the experiment. IM analyzed the data and drafted the manuscript. HN finalized the manuscript. Both authors contributed to the article and approved the submitted version.

## Conflict of Interest

The authors declare that the research was conducted in the absence of any commercial or financial relationships that could be construed as a potential conflict of interest.

## Publisher’s Note

All claims expressed in this article are solely those of the authors and do not necessarily represent those of their affiliated organizations, or those of the publisher, the editors and the reviewers. Any product that may be evaluated in this article, or claim that may be made by its manufacturer, is not guaranteed or endorsed by the publisher.

## Footnotes

1
https://www.millisecond.com

2
https://jasp-stats.org/
