# Supplementary material for: The Intention to Conceal Does Not Always Affect Time Perception
Source: Front Psychol. 2021 Dec 10;12:781685. doi: 10.3389/fpsyg.2021.781685 (PMC8702501; doi:10.3389/fpsyg.2021.781685)
Supplement: Supplementary file 1 [file Table_1.pdf]

**Supplementary Material**

**Supplementary Table 1.** Time judgement index results of the Condition × Item ANOVA in each experiment and Duration × Condition × Item ANOVA across all experiments, which were conducted separately for men and women. Results related to H1 and H2 are extracted.

| | Main effect of condition (H1) | | | | Condition × Item (H2) | | | |
|---|---|---|---|---|---|---|---|---|
| **Time judgment index** | *F* | *P* | $\eta_p^2$ | $BF_{10}$ | *F* | *p* | $\eta_p^2$ | $BF_{10}$ |
| **Men** | | | | | | | | |
| Experiment 1 [1 s, *df* = (1, 29)] | 1.978 | 0.170 | 0.064 | 0.643 | 1.000 | 0.326 | 0.033 | 0.307 |
| Experiment 2 [0.5 s, *df* = (1, 38)] | 0.001 | 0.973 | < 0.001 | 0.184 | 0.932 | 0.341 | 0.024 | 0.258 |
| Experiment 3 [2 s, *df* = (1, 30)] | 1.063 | 0.311 | 0.034 | 0.340 | 0.041 | 0.841 | 0.001 | 0.170 |
| All experiments [*df* = (1, 97)] | 0.022 | 0.883 | < 0.001 | 0.111 | 0.922 | 0.339 | 0.009 | 0.184 |
| **Women** | | | | | | | | |
| Experiment 1 [1 s, *df* = (1, 41)] | 0.265 | 0.609 | 0.006 | 0.189 | 0.001 | 0.974 | < 0.001 | 0.167 |
| Experiment 2 [0.5 s, *df* = (1, 33)] | 0.360 | 0.553 | 0.011 | 0.207 | 0.153 | 0.698 | 0.005 | 0.179 |
| Experiment 3 [2 s, *df* = (1, 39)] | 0.588 | 0.448 | 0.015 | 0.208 | 0.477 | 0.494 | 0.012 | 0.209 |
| All experiments [*df* = (1, 113)] | 0.373 | 0.543 | 0.003 | 0.113 | 0.382 | 0.538 | 0.003 | 0.123 |
